# Ceftiofur hydrochloride affects the humoral and cellular immune response in pigs after vaccination against swine influenza and pseudorabies

**DOI:** 10.1186/s12917-015-0586-3

**Published:** 2015-10-22

**Authors:** Małgorzata Pomorska-Mól, Ewelina Czyżewska-Dors, Krzysztof Kwit, Karol Wierzchosławski, Zygmunt Pejsak

**Affiliations:** Department of Swine Diseases, National Veterinary Research Institute, 24-100 Pulawy, Poland; Agrobiovet, 62-200 Gniezno, Poland

**Keywords:** Ceftiofur hydrochloride, Immune response, Vaccination, Pigs

## Abstract

**Background:**

Cephalosporins are a class of antibiotics that are active against many Gram-positive and some Gram-negative bacteria. Beyond their antibacterial activity, they are reported to have various immunomodulatory properties. It has been shown that they reduce the secretion of cytokines as well as influence the humoral and cellular immune response.

In the field conditions antibiotics are frequently administered at the same time as vaccines in pigs and, in the view of their potential immunomodulatory properties, it is important to examine their effect on the development and persistence of the post-vaccinal immune response. Ceftiofur is a very popular veterinary medicine third-generation cephalosporin with a broad spectrum of activity. It has been shown that it can inhibit cytokines secretion and in this way can potentially affect host immune response. The influence of ceftiofur on the immune response has not yet been investigated in pigs. In the present study we evaluated the influence of therapeutic doses of ceftiofur hydrochloride on the post-vaccinal immune response after vaccination with two model vaccines (live and inactivated).

**Methods:**

Seventy pigs were divided into five groups: control, unvaccinated (C), control vaccinated against swine influenza (SI-V), control vaccinated against pseudorabies (PR-V), vaccinated against SI during ceftiofur administration (SI-CEF) and vaccinated against PR during ceftiofur administration (PR-CEF). Pigs from SICEF and PR-CEF groups received therapeutic dose of ceftiofur for five days. Pigs from SI-CEF, PR-CEF, SIV and PR-V groups were vaccinated against SI and PR. Antibodies to PRV were determined with the use of blocking ELISA tests (IDEXX Laboratories, USA). Humoral responses to SIV were assessed based on haemagglutination inhibition assay. T-cell response was analyzed with the use of proliferation test. The concentrations of IFN- γ and IL-4 in culture supernatant were determined with the use of ELISA kits Invitrogen Corporation, USA).

**Results:**

The significant delay in the development of humoral response against pseudorabies virus (PRV) as well as a significant suppression of production of antibodies against swine influenza virus (SIV) was found in pigs receiving ceftiofur hydrochloride at the time of vaccination. The cellular immune response against PRV was also significantly affected by ceftiofur. In contrast, there were no significant differences between vaccinated groups with regard to the T-cell response against SIV.

From day 28 of study to day 70, the concentration of INF-γ in culture supernatants were significantly lower in group treated with ceftiofur after restimulation with PRV. While, no significant differences were observed after restimulation of PBMC with H3N2 SIV.

**Conclusions:**

The effect of an antibiotic therapy with ceftiofur hydrochloride on the humoral and cellular post-vaccinal immune responses in pigs was investigated. Ceftiofur hydrochloride was given in therapeutic doses. The results of the present study indicate that both, humoral and cell-mediated post-vaccinal immune responses can be modulated by treatment with ceftiofur hydrochloride. The results of our study point out that caution should be taken when administered this antibiotic during vaccination of pigs.

## Background

Cephalosporins are a class of antibacterial agents that are active against many Gram-positive and some Gram-negative bacteria [1–4]. Beyond their antibacterial activity, cephalosporins are reported to have various immunomodulatory properties [1, 2, 5–8]. It has been found that cephalosporins reduce the secretion of cytokines and affect migration of neutrophils [1, 2, 9, 10]. The influence of various cephalosporins on the humoral and cellular immune response has been also reported [5–8, 10]. It has been shown, that they may inhibit mitogen-induced transformation depending on the antibiotic tested, dose and the kind of mitogen used [5, 6, 10]. The effect of cephalosporins on the antibody production was also depending on compound and class of immunoglobulin tested [7, 8, 11, 12].

Because in some cases the antibiotics are administered at the same time as vaccines (i.e., after introduction of new pigs into a herd), it is important to investigate the effect of various chemotherapeutics on the development and persistence of the post-vaccinal immune response in pigs [13].

Ceftiofur is a third-generation cephalosporin developed strictly for veterinary use [1–3]. It is quite popular in veterinary medicine because of its pharmacodynamic and pharmacokinetic properties and a broad spectrum of activity against Gram-positive and Gram-negative bacteria, including β-lactamase-producing strains and anaerobes [4]. Like other cephalosporins, ceftiofur is bactericidal *in vitro*, resulting from inhibition of cell wall synthesis [14]. Ceftiofur has worldwide approvals for respiratory disease in swine (associated with *Streptococcus suis, Salmonella cholerasuis*, *Actinobacillus pleuropneumoniae* and *Pasteurella multocida*)*,* ruminants and horses and has also been approved for foot rot and metritis infections in cattle [3, 15]. The recommended dosage regimen of ceftiofur for the treatment of swine respiratory disease is 3–5 mg/kg body weight administered intramuscularly once daily for 3–5 consecutive days [4].

It has been shown previously that ceftiofur can inhibit LPS-stimulated TNF-α, IL-1β and IL-6 secretion *in vitro* via activation of the NF-κB and MAP-kinase pathways [1]. Similar results were observed during *in vivo* studies conducted on mice [2]. These data indicate that ceftiofur can affect host immune response. The influence of ceftiofur on the immune response *in vivo* is not sufficiently explained to date and has not yet been investigated in pigs.

In view of the potential immunomodulatory properties of the ceftiofur and its frequent use in pigs under field conditions, this study evaluated the influence of therapeutic doses of ceftiofur hydrochloride on the post-vaccinal immune response after vaccination with two model vaccines (live and inactivated).

## Material and methods

### Animals

Seventy pigs were bought from high health status herd, located in Lubelskie voivodeship in Poland. The owner of the herd gave the permission for use pigs in this study. The herd was seronegative for both SIV and PRV based on the routine monitoring. Pigs were transported to the animal facilities of the Polish National Veterinary Research Institute two weeks before experiment (acclimatisation period). All animals used in the experiment were confirmed negative for the antibodies against pseudorabies virus (PRV) and swine influenza virus (SIV). Only pigs that not received any of antibiotics before study were involved in the experiment. During study pigs of all groups did not receive any treatment beyond ceftiofur (in the respective groups).

Pigs were divided into five groups: control, unvaccinated (C, *n* = 10), control vaccinated against swine influenza (SI-V, *n* = 15), control vaccinated against pseudorabies (PR-V, *n* = 15), vaccinated against swine influenza during ceftiofur administration SI-CEF (*n* = 15) and vaccinated against pseudorabies during ceftiofur administration PR-CEF (*n* = 15). Animals were housed at the animal facility of National Veterinary Research Institute in independent units - one unit for the each group. Feed and water (without antibiotics) were offered *ad libitum*.

Animal use and handling protocols were approved by Local Ethical Commission (University of Life Sciences in Lublin, Poland, number: 28/2012).

### Drug and vaccines

A commercial product containing ceftiofur hydrochloride was used (Ceftiocyl, Vetoquinol Biowet Sp. z o.o.).

Two model vaccines were used for vaccination of pigs 1) vaccine against pseudorabies (Akipor 6.3, Merial, France) - as a model of live vaccine and 2) vaccine against swine influenza (GRIPOVAC, Merial, France) as a model of inactivated vaccine.

### Experimental design

Pigs from SI-CEF and PR-CEF groups received recommended dose of ceftiofur (3 mg per kg body weight per day, intramuscularly) for five days (day −1 to day 3).

Pigs from SI-CEF, PR-CEF, SI-V and PR-V groups were vaccinated intramuscularly at 10 and 12 weeks of age (0 and 14 day of study) with appropriate doses of vaccines (2 ml of each vaccine). Pigs from group C received the same dose of PBS.

Blood sampling was performed for the following indications: evaluation of antigen-specific T-cell proliferation and evaluation of humoral response. Moreover the secretion of cytokines by peripheral blood mononuclear cells (PBMC) *ex vivo* was analysed. The blood samples were taken on days: −1, 6, 9, 14, 28, 42, 56 and 70.

### Humoral response

Antibodies to the glycoprotein B (gB) and gE antigen were determined with the use of blocking ELISA tests (HerdChek*Anti-PRVgB or HerdChek*Anti-PRVgp1, IDEXX Laboratories, USA), according to manufacturer’s recommendation. Optical density (OD) was measured at 650 nm wavelength (Multiskan RC, Labsystems, Finland). The presence or absence of specific antibodies was determined by calculating the sample to negative ELISA (S/N) ratio (OD of test serum/mean OD of negative reference serum). Samples were considered to be positive for gB antigen if ELISA S/N ratio was less or equal to 0.5, while for gE antigen if ELISA S/N ratio was lower or equal to 0.6

Humoral responses to SIV hemagglutinin (H3) were assessed based on haemagglutination inhibition (HI) assay. The HI assay was performed according to the standard procedure, using 0.5 % chicken erythrocytes and 4HA units of strain H3N2 (A/Sw/Flanders/1/98). All sera were tested in serial twofold dilutions, started 1:20. For estimates of the prevalence, titres ≥ 20 were considered positive. For statistical analyses titres lower than 20 were set to 0.

### Cellular response

#### Lymphocyte proliferation assay

The proliferation assay was done at day −1, 6, 9, 14, 28, 42, 56 and 70 of study, as described previously [31]. The cells were restimulated with 50 μl of medium containing live PRV, strain NIA-3 (titer 10^6.0^ TCID_50_/50 μl) or live H3N2 SIV (A/Sw/Flanders/1/98, titer 10^6.3^ TCID_50_/50 μl and 256 hemagglutinin units). In control tubes the peripheral blood mononuclear cells were mock-stimulated or stimulated with 5 μg/ml of concanavalin (Con-A) (viability control). All samples were analysed in triplicate. The incorporated radioactivity was measured in an ultra low background liquid scintillation counter (Quantulus, PerkinElmer, USA). Proliferation was expressed as a stimulation index (S-index) calculated as follows: the number of counts per minute (cpm) for stimulated PBMCs divided by the number of cpm for the unstimulated control cells (mock-control).

Based on the S-index values observed at day −1 (before vaccination) and those observed in unvaccinated animals (the mean value plus 3 x standard deviations), the S-index equal or higher than 1.86 (PRV) or 3.66 (SIV) was considered positive for antigen-specific proliferation.

### Cytokines (IFN-γ, IL-4) secretion *in vitro*

The concentration of Th1 or Th2-type cytokines (IFN- γ and IL-4) in culture supernatant after *ex vivo* re-stimulation of PBMC with PRV and H3N2 SIV were determined with the use of ELISA kits specific for porcine IFN-γ and IL-4 (Invitrogen Corporation, USA). Unstimulated cells served as control (mock control).

PBMC were isolated and incubated (72 h) under the same conditions as for proliferation test. In each experiment, serial dilutions of swine IFN-γ and IL-4 standards were tested to determine calibration curves, which were then computer adjusted (with the use of the CurveExpert software). Concentration of cytokines in supernatants was calculated using the same software.

### Statistical analysis

A nonparametric Kruskal-Wallis test with post hoc multiple comparisons for comparison of all pairs was used (STATISTICA 8.0; StatSoft). For all analyses *p* ≤ 0.05 was considered significant.

## Results

### Humoral response against pseudorabies virus and H3N2 swine influenza virus

**Fig. 1:**
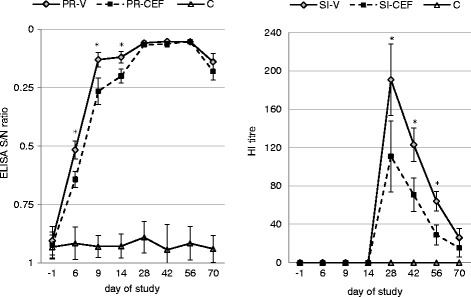
The development and persistence of antibodies specific to gB of pseudorabies virus (PRV) and H3N2 swine influenza virus (SIV). PR-V- pigs vaccinated against pseudorabies, no treatment with antibiotic. PR-CEF - pigs received ceftiofur hydrochloride during vaccination against pseudorabies. SI-V- pigs vaccinated against swine influenza, no treatment with antibiotic. SI-CEF - pigs received ceftiofur hydrochloride during vaccination against swine influenza. C - control pigs (unvaccinated, no antibiotic treatment) . * - statistically significant differences between vaccinated groups

The development and persistence of antibodies specific to gB of PRV and H3N2 SIV in animals from all groups is presented on Fig. 1.

Before vaccination all pigs had no antibodies against both gB and gE of PRV and against SIV. There were no specific antibodies against gB of PRV and against SIV in serum samples taken from unvaccinated pigs (group C).

Six days after the first vaccination in 5 out of 15 pigs from group PR-V the specific humoral response at the level considered positive was found (taking into consideration the S/N ELISA ratio). At the same time none of the pigs from PR-CEF group could be considered as positive. Starting from day 9 after the first dose of vaccine the specific antibodies to gB of PRV at the level considered positive was observed in all vaccinated pigs, however in pigs received ceftiofur the level of antibodies was significantly lower as compared to PR-V group (*p* < 0.05). The same is also true with regard to the day 14 of study. From day 28 of study no significant differences were found between ELISA S/N ratio in pigs from PR-CEF and PR-V group (*p* > 0.05). In contrast significant differences were observed between vaccinated and unvaccinated groups from day 6 of study to the end of experiment (*p* < 0.05).

None of the unvaccinated pigs had antibodies against SIV at the end of the study. No seroconversion was observed in vaccinated pig after the first vaccination (Fig. 1). In general, antibody levels peaked 2 weeks after second vaccination, after which they steadily decreased. From day 28 of study to day 56 the mean antihemagglutinin 3 (Anti-HA3) antibody titer were significantly higher in pigs from group SI-V (*p* < 0.05). Moreover, at day 70 of study, 7 out of 15 pigs from SI-CEF group were seronegative to H3N2 SIV, while in group SI-V only 2 out of 15 animals had Anti-HA3 antibody titer below 20.

### Cellular response against pseudorabies virus and H3N2 swine influenza virus

**Fig. 2:**
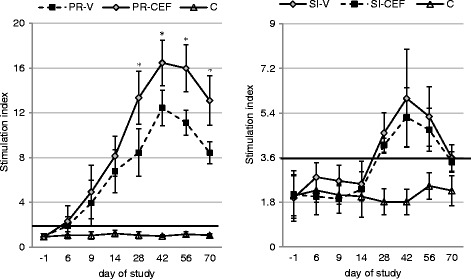
The mean stimulation index values observed in pigs after vaccination against pseudorabies and swine influenza and in control, not vaccinated animals. The bold lines indicate border-line between nonspecific and antigen-specific proliferation. PR-V- pigs vaccinated against pseudorabies, no treatment with antibiotic. PR-CEF - pigs received ceftiofur hydrochloride during vaccination against pseudorabies. SI-V- pigs vaccinated against swine influenza, no treatment with antibiotic. SI-CEF - pigs received ceftiofur hydrochloride during vaccination against swine influenza. C - control pigs (unvaccinated, no antibiotic treatment). * - statistically significant differences between vaccinated groups

The mean S-index values after vaccination against PRV and H3N2 SIV and in control, not vaccinated animals are shown on Fig. 2. In unvaccinated group the SI values ranged from 0.67 to 1.53 (for PRV) and from 1.14 to 2.91 (for SIV) during the period of study.

The PRV- specific proliferation was observed 6 days after the first dose of vaccine in 9 out of 15 pigs from PR-V group and in 3 out of 15 pigs from PR-CEF group. However, at this time-point no significant differences between vaccinated and unvaccinated groups were observed with regard to the mean value of S-index (*p* > 0.05). Starting from day 9 of study the PRV-specific proliferation was observed in all vaccinated pigs. The mean value of S-index was significantly higher in pigs from PR-V group as compared to pigs from PR-CEF from day 28 to the end of study (*p* < 0.05).

No SIV-specific proliferation was observed in pigs before booster dose of vaccine. Two weeks after the second dose of vaccine an antigen-specific proliferation was found in each vaccinated pig. The mean value of S-index did not differ significantly between vaccinated groups (*p* > 0.05) during the period of study. However, starting from day 28 of study (2 weeks after the second dose of vaccine), the significant difference between S-index in vaccinated and unvaccinated pigs was noted (*p* < 0.05).

### Th1- and Th2-type cytokine secretion following *in vitro* stimulation of PBMC

**Fig. 3:**
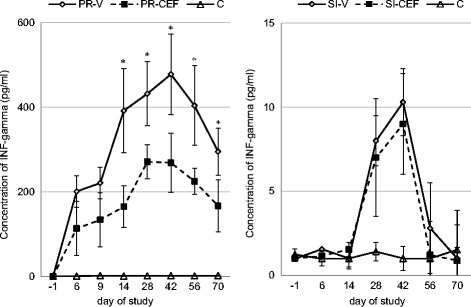
Interferon-gamma (IFN-γ) secretion by peripheral blood mononuclear cells. The points represent the mean concentration of IFN-γ in culture supernatant after stimulation with pseudorabies virus (PRV) or swine influenza virus (SIV). PR-V- pigs vaccinated against pseudorabies, no treatment with antibiotic. PR-CEF - pigs received ceftiofur hydrochloride during vaccination against pseudorabies. SI-V- pigs vaccinated against swine influenza, no treatment with antibiotic. SI-CEF - pigs received ceftiofur hydrochloride during vaccination against swine influenza. C - control pigs (unvaccinated, no antibiotic treatment). * - statistically significant differences between vaccinated groups

The summary of INF- γ analysis is shown in Fig. 3. The mean constitutive production of INF-γ (without PRV or H3N2 SIV stimulation) in experimental pigs did not exceed 9.91 pg/ml. After *in vitro* exposure to live PRV or H3N2 SIV, naïve PBMC never secreted INF-γ higher than 12.52 pg/ml and 13.78 pg/ml, respectively. In unvaccinated group (C) there was no significant increase of INF-γ concentration after PRV and H3N2 SIV restimulation in all sampling points.

In contrast, *in vitro* PRV stimulation of PBMC from vaccinated animals resulted in high amounts of INF-γ in the culture supernatant. From day 28 of study to day 70, the concentration of INF-γ in culture supernatants were significantly higher in pigs from PR-V group as compared with pigs from PR-CEF group. Significant differences between vaccinated and unvaccinated pigs were observed from day 9 to the end of study (*p* < 0.05).

In contrast, no significant differences were observed between groups vaccinated against swine influenza after restimulation of PBMC with H3N2 SIV (*p* > 0.05).

However, in pigs from both vaccinated groups the significantly higher production of INF- γ by PBMC was observed after H3N2 SIV from day 28 to day 70 of study as compared with control pigs (C)

*In vitro* stimulation with both viruses did not induce measurable secretion of IL-4 by PBMC in the case of both vaccinated and unvaccinated animals. In supernatants from stimulated and unstimulated cultures the level IL-4 was undetectable (<15.6 pg/ml).

## Discussion

The present study investigated the influence of ceftiofur hydrochloride on the humoral and cellular post-vaccinal immune response in pigs. The effect of simultaneous administration of antibiotics and vaccines is frequently underestimated when applying immunoprophylaxis programs in the field conditions. Without exact knowledge of the effects of antibiotics on the post-vaccinal response they are frequently applied at the time of vaccination. To the authors best knowledge the effect of ceftiofur hydrochloride on the post-vaccinal response against pseudorabies and swine influenza have not been previously studied. In the present study, we have established that the immune response produced with PRV and SIV vaccination can be affected by the treatment with ceftiofur hydrochloride when applying at the same time as vaccines.

Various immunomodulatory properties have been reported previously for several cephalosporins, including ceftiofur [1, 2, 9, 10, 12, 16]. In the present study a delay in the development of humoral response against PRV was found in pigs receiving ceftiofur hydrochloride when compared with PR-V group. The significant reduction in ELISA S/N ratio against gB PRV antigen was observed before the second dose of vaccine. It may suggest that ceftiofur hydrochloride exert its effect mainly on the IgM isotype. However, it is worth to mention that antibody-response returned to normal level after the boost. Moreover, a significant reduction in the development of post-vaccinal Anti-HA3 antibody was noted in pigs from SI-CEF group.

The influence of cephalosporins (cefotaxime, cefodizime, ceftiofur sodium) on the humoral immunity was also observed by others in various animal species [1, 2, 9, 10, 12, 17]. Pulverer [16] reported that in mice treated with therapeutic doses of cefotaxime, but not with cefodizime, a remarkable and long-lasting inhibition of the IgM antibody production has been found. Similar trends have been seen concerning the IgG antibody response. In opposite, Borowski et al. [12], reported that cephradine, in contrast to cefotaxime, suppressed the humoral response in mice in doses corresponding to those used for the treatment of patients. The results reported to date indicate that various cephalosporins can produce different effects on the immune response.

The marked reduction in the Anti-HA3 antibody titer may be of great importance since Anti-HA antibody has an important role in the protection against swine influenza [18, 19]. In pigs vaccinated during antibiotic therapy the mean HI titre were significantly lower than in pigs not receiving ceftiofur. In addition, six weeks after the second dose of vaccine all pigs from SI-V group had HI titers exceeding the level commonly associated with protective immunity against influenza (HI titre 40) [18]. In SI-CEF group only 7 out of 15 pigs had HI titre 40 at that time. At the end of study the HI titers equal or over 40 were observed only in pigs from SI-V group. Vaccination of pigs against SIV is an important tool used to prevent and control the disease in pigs. However, the results of our study indicate that administration of vaccine during treatment with ceftiofur hydrochloride can have a negative effect on the development and persistence of post-vaccinal humoral immunity against SIV and in this way affect the protection against infection.

Simultaneously to reduction of humoral response in ceftiofur-treated pigs, no significant influence on the cellular immunity against SIV was observed in our investigation. In the current experiment the proliferation assay has been used to evaluate the antigen-specific cellular immune response [20–22]. A high level of proliferation in response to antigen correlates with the expansion of antigen-specific lymphocytes and indicates the superior anamnestic responses of memory cells [23]. Moreover, IFN-γ production has been reported to be a marker of cellular immunity in some viral infections of pigs [20, 24].

Our results showed that, the vaccinated ceftiofur-treated group had a significant decrease in IFN-γ production above non-treated group after PRV stimulation. Moreover, after PRV restimulation of PBMC isolated from blood of pigs belonging to PR-CEF group the significantly lower values of S-index were observed as compared with PR-V group (*p* < 0.05). In contrast, no significant differences in IFN-γ secretion were found after stimulation of PBMC with H3N2 SIV. No significant differences regarding S-index between SIV vaccinated groups were also noted.

The negative impact of ceftiofur hydrochloride on the IFN-γ secretion after PRV restimulation can potentially be important from a clinical point of view. It is well documented that cytokines secreted by T lymphocytes, play a crucial role in the initiation and maintenance of antiviral immune responses [25]. The Th1-type immune response is supposed to limit viral replication during pseudorabies infection [25], thus lower production of IFN-γ may be a sign of worst antiviral activity against PRV. In contrast, during influenza infection, the cell-mediated immunity plays a role mostly in the recovery from influenza infection. It does not seem to contribute notably in preventing infection [26]. The anti-HA antibody, the development and persistence of which were negatively influenced by ceftiofur, are the most important in neutralizing the virus, and thus prevention of the disease [26].

Ci et al. [2] have shown that pre-treatment with a dose of ceftiofur that prevented death in mice, significantly decreases TNF-α, IL-6 and IL-1 production. The influence of ceftiofur on the proportions among lymphocytes subset in lymphatic organs has been also reported in chicks by Chrząstek & Wieliczko [27]. The authors concluded that treatment with ceftiofur might have an impact on the immune response in chickens.

The mechanism of inhibition of T-cell response against PRV by ceftiofur remains to be clarified. However the influence of ceftiofur on the production and secretion of mediators of the immune response (i.e., cytokines) can be considered as the probable reason [1, 2, 27, 28].

The negative impact of other cephalosporins (cefotaxime) on T-cell immune response has been also reported by Pulverer [16], who has found that cefotaxime induced a significant and long-lasting inhibition of cellular immunity in mice. The same results have been also reported by others [29, 30]. In contrast, no effects on the cellular immunity were observed for cefodizime [16]. The significant suppression of *in vitro* lymphocyte responses to the mitogens by various cephalosporins (cephalexin, cephradine, cephalotin) has also been reported [6]. Cephalexin and cephradine suppress lymphocyte response to PHA (phytohemagglutinin), ConA and PWM (pokeweed mitogen), suggesting that these cephalosporins are probably acting by mechanism other than a specific interference with mitogen binding cell membrane [6]. In the study conducted by Borowski et al. [12] the *in vitro* response of mouse splenocytes to PHA was suppressed by cephradine and cephodizime, however this occurred at therapeutic concentrations only in the case of cephradine. Neither cephalosporin affected the phagocytic and chemotactic activity of mouse peritoneal macrophages and rabbit microphages [12].

## Conclusion

In conclusion, the effect of an antibiotic therapy with therapeutic doses of ceftiofur hydrochloride on the humoral and cellular post-vaccinal immune responses in pigs was investigated. The results of the present study indicate that both, humoral and cell-mediated post-vaccinal immune responses can be modulated by treatment with therapeutic doses of ceftiofur hydrochloride. However the impact of ceftiofur hydrochloride on the humoral immunity seems to be related to the vaccine. Because only one vaccine of each class has been use we cannot exclude that the impact of ceftiofur hydrochloride may also depend on the vaccine. The results of our study confirmed that caution should be taken when administered this antibiotic during vaccination of pigs.

## References

[1] Ci X, Song Y, Zeng FQ, Zhang XM, Li HY, Wang XR (2008). Ceftiofur impairs proinflammatory cytokine secretion through the inhibition of the activation of NF-kappaB and MAPK. Bochem Biophys Res Commun.

[2] Ci X, Li HY, Song Y, An Y, Yu Q, Zeng F (2008). Ceftiofur regulates LPS-induced production of cytokines and improves LPS-induced survival rate in mice. Inflammation.

[3] Hornish RE, Kotarski SF (2002). Cephalosporins in veterinary medicine - ceftiofur use in food animals. Curr Top Med Chem.

[4] Tantituvanont A, Yimprasert W, Werawatganone P, Nilubo D (2009). Pharmacokinetics of ceftiofur hydrochloride in pigs infected with porcine reproductive and respiratory virus. J Antimicrob Chemother.

[5] Banck G, Forsgren A. Antibiotics and suppression of lymphocyte function *in vitro*. Antimicrob Agents Chemother. 1979;16:554–60. 10.1128/aac.16.5.554 PMC352904 316685

[6] Chaperon EA, Sanders WE (1978). Suppression of lymphocyte responses by cephalosporins. Infect Immun.

[7] Akahane K, Furuhama K, Kato M, Une T, Onodera T (1990). Influences of cephem antibiotics on the immune response in mice. Chemotherapy.

[8] Furuhama K, Benson RW, Knowles BJ, Roberts DW (1993). Immunotoxicity of cephalosporins in mice. Chemotherapy.

[9] Meloni F, Ballabio P, Bianchi L, Grassi FA, Gialdroni Grassi GG. Cefodizime modulates *in vitro* tumor necrosis factor-alpha, interleukin-6 and interleukin-8 release from human peripheral monocytes. Chemotherapy. 1995;41:289–95. 10.1159/000239358 7555210

[10] Forsgren A, Banck G. Influence of antibiotics on lymphocyte function *in vitro*. Infection. 1978;6 Suppl 1:91–7.

[11] Gillissen G (1981). Nouveaux antibiotiques beta-lactam et reponse immunitaire. Med Hyg.

[12] Borowski J, Jakoniuk P, Talarczyk J (1985). The influence of some cephalosporins on immunological response. Drugs Exp Clin Res.

[13] Pomorska-Mól M, Pejsak Z. Effects of antibiotics on acquired immunity *in vivo* – current state of knowledge. Pol J Vet Sci. 2012;15:583–8. 10.2478/v10181-012-0089-0 23214384

[14] Brown SA, Hanson BJ, Mignot A, Millérioux L, Hamlow PJ, Hubbard VL (1999). Comparison of plasma pharmacokinetics and bioavailability of ceftiofur sodium and ceftiofur hydrochloride in pigs after a single intramuscular injection. J Vet Pharmacol Ther.

[15] Halbur P, Thanawongnuwech R, Brown G, Kinyon J, Roth J, Thacker E (2000). Efficacy of antimicrobial treatments and vaccination regimens for control of porcine reproductive and respiratory syndrome virus and Streptococcus suis coinfection of nursery pigs. J Clin Microbiol.

[16] Pulverer G (1992). Effects of cefodizime and cefotaxime on cellular and humoral immune responses. Infection.

[17] Limbert M, Bartlett RR, Dickneite G, Klesel N, Schorlemmer HU, Seibert G (1984). Cefodizime, an aminothiazolyl cephalosporin. IV. Influence on the immune system. J Antibiotics.

[18] Coudeville L, Bailleux F, Riche B, Megas F, Andre P, Ecochard R (2010). Relationship between haemagglutination-inhibiting antibody titres and clinical protection against influenza: development and application of a bayesian random-effects model. BMC Med Res Methodol.

[19] Markowska-Daniel I, Pomorska-Mól M, Pejsak Z (2011). The influence of age and maternal antibodies on the postvaccinal response against swine influenza viruses in pigs. Vet Immunol Immunopathol.

[20] Van Rooij EMA, de MGM B, de YE V, Middel WGJ, Boersma WJA, Bianchi ATJ (2004). Vaccine-induced T-cell mediated immunity plays a critical role in early protection against pseudorabies virus (suid herpes virus type 1) infection in pigs. Vet Immunol Immunopathol.

[21] Kimman TG, De Bruin TM, Voermans JJ, Peeters BP, Bianchi AT (1995). Development and antigen specificity of the lymphoproliferation responses of the pigs to pseudorabies virus: dichotomy between secondary B- and T-cell responses. Immunology.

[22] Pomorska-Mól M, Markowska-Daniel I (2010). Interferon-γ secretion and proliferative responses of peripheral blood mononuclear cells after vaccination of pigs against Aujeszky’s disease in the presence of maternal immunity. FEMS Immunol Med Microbiol.

[23] Sandbulte MR, Roth JA (2004). Methods for analysis of cell-mediated immunity in domestic animals species. J Am Vet Med Assoc.

[24] Hoegen B, Saalmüller A, Röttgen M, Rziha HJ, Geldermann H, Reiner G (2004). Interferon-gamma response of PBMC indicates productive pseudorabies virus (PRV) infection in swine. Vet Immunl Immunopathol.

[25] Fischer T, Büttner M, Rziha HJ (2000). T helper 1-type cytokine transcription in peripheral blood mononuclear cells of pseudorabies virus (*Suid herpesvirus 1*)-primed swine indicates efficient immunization. Immunology.

[26] Cox RJ, Brokstad KA, Ogra P (2003). Influenza virus: Immunity and vaccination strategies. Comparison of the immune response to inactivated and live, attenuated influenza vaccines. Scand J Immunol.

[27] Chrząstek K, Wieliczko A (2015). The influence of enrofloxacin, florfenicol, ceftiofur and E. coli LPS interaction on T and B cells subset in chicks. Vet Res Commun.

[28] Schroecksnadel K, Winkler C, Werner ER, Sarcletti M, Romani N, Ebner S, et al. Interferon-gamma-mediated pathways and *in vitro* PBMC proliferation in HIV-infected patients. Biol Chem. 2009;390:115–23. 10.1515/BC.2009.018 19040353

[29] Roszkowski W, Ko HL, Roszkowski K, Jeljaszewicz J, Pulverer G (1985). Effect of selected antibiotics on the cellular and humoral immune response in mice. Zentralbl Bacteriol.

[30] Roszkowski W, Ko HL, Roszkowski K, Jeljaszewicz J, Pulverer G (1985). Antibiotics and immunomodulation: effects of cefotaxime, amikacin, mezlocilin, piperacilin and clindamycin. Med Microbiol Immunol.

[31] Pomorska-Mól M, Markowska-Daniel I, Rachubik J (2012). Development of early humoral and cell-mediated immunity in piglets with experimentally induced subclinical swine influenza. Bull Vet Inst Pulawy.

